# Toward Understanding the Genetic Basis of Yak Ovary Reproduction: A Characterization and Comparative Analyses of Estrus Ovary Transcriptiome in Yak and Cattle

**DOI:** 10.1371/journal.pone.0152675

**Published:** 2016-04-04

**Authors:** Daoliang Lan, Xianrong Xiong, Cai Huang, Tserang Donko Mipam, Jian Li

**Affiliations:** 1 College of Life Science and Technology, Southwest University for Nationalities, Chengdu ic610041, Peoples’ Republic of China; 2 Institute of Qinghai-Tibetan Plateau, Southwest University for Nationalities, Chengdu, 610041, Peoples’ Republic of China; Wageningen UR Livestock Research, NETHERLANDS

## Abstract

**Background:**

Yaks (*Bos grunniens*) are endemic species that can adapt well to thin air, cold temperatures, and high altitude. These species can survive in harsh plateau environments and are major source of animal production for local residents, being an important breed in the Qinghai–Tibet Plateau. However, compared with ordinary cattle that live in the plains, yaks generally have lower fertility. Investigating the basic physiological molecular features of yak ovary and identifying the biological events underlying the differences between the ovaries of yak and plain cattle is necessary to understand the specificity of yak reproduction. Therefore, RNA-seq technology was applied to analyze transcriptome data comparatively between the yak and plain cattle estrous ovaries.

**Results:**

After deep sequencing, 3,653,032 clean reads with a total of 4,828,772,880 base pairs were obtained from yak ovary library. Alignment analysis showed that 16992 yak genes mapped to the yak genome, among which, 12,731 and 14,631 genes were assigned to Gene Ontology (GO) categories and Kyoto Encyclopedia of Genes and Genomes (KEGG) pathways. Furthermore, comparison of yak and cattle ovary transcriptome data revealed that 1307 genes were significantly and differentially expressed between the two libraries, wherein 661 genes were upregulated and 646 genes were downregulated in yak ovary. Functional analysis showed that the differentially expressed genes were involved in various Gene Ontology (GO) categories and Kyoto Encyclopedia of Genes and Genomes (KEGG) pathways. GO annotations indicated that the genes related to “cell adhesion,” “hormonal” biological processes, and “calcium ion binding,” “cation transmembrane transport” molecular events were significantly active. KEGG pathway analysis showed that the “complement and coagulation cascade” pathway was the most enriched in yak ovary transcriptome data, followed by the “cytochrome P450” related and “ECM–receptor interaction” pathways. Moreover, several novel pathways, such as “circadian rhythm,” were significantly enriched despite having no evident associations with the reproductive function.

**Conclusion:**

Our findings provide a molecular resource for further investigation of the general molecular mechanism of yak ovary and offer new insights to understand comprehensively the specificity of yak reproduction.

## Introduction

Yaks *(Bos grunniens*), known as the “plateau ship,” are endemic species distributed mainly in the Qinghai–Tibet Plateau and the adjacent alpine or subalpine regions in China; yaks are also the only bovine animal known to live in the highest altitude regions (average altitude of 3,000 meters above sea level) worldwide [1, 2]. These species can adapt well to the alpine grassland environment, but they can also thrive and reproduce under harsh plateau environmental conditions, such as thin air, cold temperatures, and short grass [3]. Yak is the typical representative animal to learn the adaptability to the highland environment, and its specific physiological mechanisms are stable and heritable after long-term adaption and evolution in this environment [4]. Also, to study on the physiological mechanisms of yaks under special plateau conditions should be of great significance to learn the influences of the high-altitude environment to the physiological mechanisms [1].

Yaks provide milk, meat, wool, service force, fuel, and other daily necessities for local pastoralists, making them an important breed in the plateau region [1,5, 6]. However, yaks reach sexual maturity more slowly and generally have lower fertility compared with ordinary cattle that live in the plains [7, 8]. The average reproduction rate of an adult yak is only 48.61%, of which more than half represent one birth in two years or two births in three years. Moreover, the estrus rate of female yak is low, and more than 90% of postpartum female yaks cannot be rutted during the estrus season of the same year [9, 10].

Ovary is an important reproductive organ in female mammals. Its many functions include providing fertile oocytes, secreting reproductive hormones, and maintaining estrus cycles of female animals. Ovary function directly influences the fecundity of female animals [11]. During each estrus cycle, the ovary undergoes proliferation, invasion, differentiation, and cell apoptosis; these normal physiological changes directly affect and/or determine the ovulation, fertilization rate, and the litter size of female animals [12]. Compared with other cattle, the yak ovary is smaller, the ovary mesentery is shorter, and the position is relatively fixed, but the overall structure is similar [13]. To date, studies about yak ovary mainly focus on its shape and anatomy. However, the molecular basis of the yak ovary is poorly characterized and its molecular mechanism remains unknown.

The implementation of ovarian function is a complex process that involves the transcriptional regulation of a large number of genes; moreover, the divergence of gene expression is an important component of species evolution and an essential means to generate biological diversity [12, 14]. Therefore, a transcriptome study is needed to understand the molecular mechanism of yak ovary and the specificity of yak reproduction. With the recent developments in high-throughput sequencing technologies, transcriptome sequencing (RNA sequencing or RNA-seq) has provided a powerful tool for large-scale transcriptome studies and is highly advantageous over conventional methods [12]. Moreover, the recent completion of the yak genomic sequence also allows further transcriptome study [1]. In the present study, RNA-seq technology was applied for the comparative analysis of transcriptome data between yak and plain cattle estrus ovaries. The RNA-seq technology can provide insight into the genome-wide divergence of expression patterns between these ovaries during the estrus cycle. The results of this study may serve as a basis to investigate the general molecular mechanism of the yak ovary and may provide an initial step to understand the specificity of yak reproduction comprehensively.

## Methods and Materials

### Sample collection

Identification of yak estrus was performed as described by Quan and Zi et al. [15, 16]. Three healthy 4-year-old female Maiwa yaks in their natural estrus cycles and with similar body sizes were randomly selected from a plateau slaughter farm (Hongyuan, Sichuan, China, 31°51′ N to 33°19′ N and 101°51′ E to 103°23′ E; average altitude of 3,600 meters above sea level). Similarly, three healthy 4-year-old female yellow cattle (*Bos taurus domesticus*) in their natural estrus were randomly selected from a slaughter farm in the plains (Chengdu, Sichuan, China, 30°05′ N to 31°26′ N and 102°54′ E to 104°53′ E; average altitude of 750 meters above sea level). The ovarian tissues of the yak and ordinary cattle were collected immediately after slaughtering and were observed. Afterward, the tissues were frozen in liquid nitrogen for RNA extraction. This study was approved by the ethics committee of the Southwest University for Nationalities (Chengdu, China). Approval from the animal use and care committee was not required for this study because the samples were obtained from government-inspected slaughter facilities.

### RNA extraction, cDNA library construction, and Illumina sequencing

The total RNA of the same parts of ovarian tissues obtained from the yak and cattle was extracted using a TRIzol reagent (Life Technologies, USA) according to the manufacturer’s instructions. Each extracted RNA sample was treated with RNase-free DNAse I (Takara [Dalian], Japan) to remove potential genomic DNA contamination. Two RNA pools were prepared by mixing equal quantities of three RNA samples per yak and ordinary cattle group. Subsequently mRNA purification and cDNA sequencing library preparation were performed with the TruSeq RNA Sample Prep Kit (Illumina, USA) following the manufacturer’s instructions. In brief, the mRNA was purified using oligo-dT attached magnetic beads and fragmented (about 200 bp) using divalent cations under elevated temperature and then applied as a template for first-strand cDNA synthesis. Second-strand cDNA was then synthesized using RNase H, dNTP, and DNA polymerase I. After purification and paired-end (PE) repair, the cDNA fragments were ligated to sequencing adapters and amplified by polymerase chain reaction (PCR) to obtain the final PE library. After quality-control (QC) tests using an Agilent 2100 Bioanalyzer and an ABI StepOnePlus real-time PCR system, the paired-end of library was sequenced with a read length of 90 bp on the Illumina HiSeq 2000 platform.

### Transcriptome data analysis

The raw reads produced by Hiseq 2000 sequencing were subjected to QC tests. Afterward, the raw reads were filtered into clean reads by removing adaptor sequences,reads with unknown bases call more than 5% and low-quality reads (<Q20) using Blat software [17]. The clean reads were aligned to the yak genome reference sequences (version 1.0) [18] using SOAPaligner/SOAP2 software[19]. The alignment data were then utilized to calculate the distribution of reads on reference genes and perform coverage analysis. The gene expression levels were calculated using the RPKM method (reads per kilobase transcriptome per million mapped reads). The differentially expressed genes (DEGs) between yak and cattle libraries were identified by a rigorous algorithm developed by BGI based on “the significance of digital gene expression profiles” [20], and the method used is described as follow:Denote the number of unambiguous clean tags (which means reads in RNA_Seq) from gene A as x, given every gene's expression occupies only a small part of the library, x yields to the Poisson distribution:

$$P(x)=\frac{{\text{e}}^{-\lambda }\lambda^{x}}{x!}(\lambda \;\mathrm{is the real transcripts of the gene})$$

The total clean tag number of the sample 1 is N 1, and total clean tag number of sample 2 is N 2; gene A holds x tags in sample 1 and y tags in sample 2. The probability of gene A expressed equally between two samples can be calculated with:

$$2\sum_{i=0}^{i=y}p(i|\;x)$$

$$\text{or}\;2\times (1-\sum_{i=0}^{i=y}p(i|x))(if\sum_{i=0}^{i=y}p(i|x)>0.5)$$

$$p(y|x)={\left(\frac{N_{2}}{N_{1}}\right)}^{y}\frac{(x+y)!}{x!y!{\left(1+\frac{N_{2}}{N_{1}}\right)}^{\left(x+y+1\right)}}$$

We used a P-value corresponding to a differential gene expression test at statistically significant levels. False discovery rate (FDR) ≤0.001 and the absolute value of log2Ratio≥1 were employed as the threshold to determine the significance of gene expression difference[21]. The function of DEGs was annotated by the GO and KEGG pathway enrichment analyses at a P ≤0.05.

### Verification by real-time PCR (qRT-PCR)

DEGs were detected using qRT-PCR to confirm the sequencing data. Information on the individual primer sequences of the 15 randomly selected DEGs is listed in S1 Table. The total RNA of the yak and cattle ovary tissues sampled during the same phase was extracted using a TRIzol reagent (Life Technologies, USA). The concentration of each RNA sample was adjusted to 1 μg/μL using nuclease-free water, and 2 μg of total RNA was reverse transcribed in a 20 μL reaction system using the SuperScript® III First-Strand Synthesis System (Life Technology, USA). For qRT-PCR, a SYBRHPremix Ex TaqTM II (Tli RNaseH Plus) Kit (Takara, Japan) and an ABI7500 FAST real-time PCR system (ABI) were used according to the manufacturers’ instructions. Reaction conditions were as follows: 95°C for 1 min, followed by 40 cycles of 95°C for 10 s, and 60°C for 40 s. A final solubility curve analysis was completed. β-Actin was used as an internal control gene. The relative expression of each gene was calculated based on a previously described 2^−ΔΔCt^ method[22]. In addition, 5 genes were randomly selected from the above 15 DEGs, and the expressions of the 3 individuals from the pooled samples of yak and cattle were tested by qRT-PCR for variation detection between group members.

## Results and Discussion

### Genetic architecture of the yak ovary transcriptome

For elaborate the general physiological molecular features of the estrous ovary of yaks, we established yak and cattle ovary transcriptome library by pooled samples respectively. The variation detection results between group members indicated that the gene expressions of the 3 individuals of the pooled samples were basically similar(S2 Table), which proved the feasibility and consistency of the pooled samples in this study. The transcriptome sequencing data were deposited in the NCBI Sequence Read Archive database (accession number: SRX335453 and SRX1302580). After the removal of low-quality reads (i.e., reads containing only adaptors, adaptor sequences, and empty reads), we obtained 53,653,032 clean reads with 4,828,772,880 base pairs, as well as 54,855,396 clean reads with 4,936,985,640 base pairs in the yak and cattle library, respectively. The analyses of base composition and quality showed that the reads exhibited balanced base compositions and that the ratios of Q20 (i.e., the quality of bases ≥ 20) in yak and cattle library were 94.7% and 94.5%, respectively. This result indicates successful library construction and good sequencing quality. Alignment analysis showed that 33,168,751 (61.82%) of the total yak clean reads and 31,730,973 (57.84%) of the total cattle clean reads were mapped to the yak genome. Gene coverage statistics showed that 16,992 and 16,904 yak genes were mapped in yak and cattle ovary library, respectively. For the elaborate the basic molecular features of the ovary of yaks and convenience of comparison, we used yak genomes as the reference genome. The transcriptome pattern was similar between yak and cattle ovary; this basic sequencing data suggest that the yak and cattle genes were highly similar, which agrees with a previous study in yak genome [1]. In addition, a large proportion of reads (about 30%) that could not be mapped to existing genes were found, but these reads were located between them in the existing genome. Some (if not all) of these reads may be new transcripts or genes that can provide useful basis for novel genomic information on yak.

The functions of the expressed gene profiles of yak ovary were further annotated by GO and KEGG analyses. Analysis of the GO annotations showed that 12731 of the mapped genes were annotated in 59 categories under biological process, cellular component, and molecular function (S1 Fig). In the biological process category, most of the annotated genes were involved in “cellular process”, followed by “single-organism process” and “metabolic process”. In addition, 7068 genes were associated with development and reproduction, namely, “developmental and reproductive process”, “reproduction”, and “reproductive process”. These results are consistent with the biological characteristics and function of the ovary. In the cellular component category, most of the genes were involved in “cell”, followed by “cell part” and “organelle”. In the molecular function category, most of the genes were involved in “binding”, followed by “catalytic activity” and “molecular transducer activity”. Previous microarray studies showed that the RNA-binding molecular function category accounted for a large proportion in the expressed genes in bovine oocytes[23, 24]; thereby confirming the results of the present study that “binding” plays an important role in the normal physiological activities of the yak ovary. KEGG analysis showed that 14631 mapped genes were involved in 258 pathways. Among top 10 pathways (S3 Table), “focal adhesion” was the most enriched, followed by “pathways in cancer” and “extracellular matrix (ECM)-receptor interaction”. However, further studies are needed to determine the exact functions of these pathways in the yak ovary.

### Analysis of DEGs

Comparison between yak and cattle ovary transcriptome data revealed that 1307 genes were differentially expressed between the two libraries, in which 661 genes were upregulated and 646 were downregulated. To validate these gene expression data, we selected 15 DEGs to perform qRT-PCR on the yak and cattle ovary tissues sampled during the same phase. We found that the expression patterns of these DEGs using qRT-PCR revealed similar variation trends with the transcriptome data (S1 Table), indicating that our results were reliable. To gain insight into the biological implications of DEGs, we performed GO and KEGG enrichment analyses for the DEGs. GO enrichment analysis showed that the DEGs were enriched in many GO categories distributed among biological processes, cellular components, and molecular functions.

In the biological process category, 3,067 GO categories were enriched, and the top 10 of which are shown in Table 1. Among the top 10 GO categories, three GO categories on adhesion including “biological adhesion,” “cell adhesion,” and “cell–cell adhesion” were most enriched. Previous studies showed that the follicles of ovaries do not have a micro-environment vascular system. A wide gap connection exists between oocytes, cumulus cells, and other follicular cells, thereby forming a complete functional joint venture. An oocyte mainly communicates with its surrounding cells (granulose and theca cells) through cell adhesion and connection [25, 26]. The result indicates that adhesion through differential cell connections may exist between the yak and cattle ovary in the estrus cycle. In addition, two GO categories related to hormones, namely, “response to steroid hormone stimulus” and “response to estrogen stimulus,” also screened in the top 10 GO categories. As expected, the hormones play an important role in regulating the female animal reproductive function. In these two GO categories, we found that the estrogen-receptor beta (ERβ) gene was significantly downregulated in yak ovary. ERβ, which contains the most number of estrogen receptors, is located in the granulosa or follicular cells during growth period and in some mesenchymal cells [27]. According to previous studies, the expression of ERβ increases along with the development of follicles; the ERβ knockout mouse model demonstrated a significant increase of follicular atresia and a decrease of ovulation [28, 29]. This result suggests that ERβ plays an important role in follicular development and is directly involved in ovulation. In this study, the expression of the ERβ gene significantly declined in yak ovary compared with that in the cattle ovary, indicating that a different mechanism about hormone regulation may exists in yak ovaries.

**Table 1:** Top 10 enrichment GO categories in the biological processes of DGEs.

Gene Ontology term	Cluster frequency	Genome frequency	Corrected P-value
biological adhesion	91 out of 965 genes^a^, 9.4%	715 out of 15573 genes^b^, 4.6%	8.88e-08
cell adhesion	90 out of 965 genes, 9.3%	711 out of 15573 genes, 4.6%	1.54e-07
cell–cell adhesion	44 out of 965 genes, 4.6%	312 out of 15573 genes, 2.0%	0.00078
anatomical structure development	312 out of 965 genes, 32.3%	3964 out of 15573 genes, 25.5%	0.00139
anatomical structure morphogenesis	170 out of 965 genes, 17.6%	1915 out of 15573 genes, 12.3%	0.00142
developmental process	345 out of 965 genes, 35.8%	4472 out of 15573 genes, 28.7%	0.00182
response to steroid hormone stimulus	47 out of 965 genes, 4.9%	362 out of 15573 genes, 2.3%	0.00355
response to estrogen stimulus	28 out of 965 genes, 2.9%	170 out of 15573 genes, 1.1%	0.00577
response to xenobiotic stimulus	22 out of 965 genes, 2.3%	128 out of 15573 genes, 0.8%	0.03572
glutathione metabolic process	12 out of 965 genes, 1.2%	45 out of 15573 genes, 0.3%	0.03925

a. The number of differentially expressed genes with biological processes with GO annotation.

b. The number of genes in the entire yak genome with biological processes with GO annotation.

In the molecular functions category, 667 GO categories were enriched, and the top 10 significantly enriched GO categories are shown in Table 2. Among the top 10 GO categories, the most enriched was the “calcium ion binding.” Calcium ions, as second intracellular messengers, regulate many important physiological and pathological processes in cells. The regulation of growth and meiosis of mammalian oocytes, in addition to the participation of hormones and growth factors, among others, calcium ions are also closely associated with the regulation of meiotic maturation of ooctyes [30, 31]. The “calcium ion binding” category is the most enriched in this study, suggesting that the regulation of meiotic maturation of ooctyes may exist differently between yak and cattle ovaries. Notably, the cation transmembrane transport-related GO categories possessed a considerable portion (6/10) in the top 10 enriched GO categories. The ion activity of cell membranes is closely associated with the regulation of intracellular environment, such as pH, osmotic pressure, nutrient absorption, and membrane potential; it also plays an important role in the regulation of neurotransmitter secretion and hormone metabolism [32]. In addition, the membrane concentration gradient established by the ion transmembrane transport provides the energy source for a series of cell metabolic activities. In many cation transmembrane transport activities, the transport of calcium and zinc ions plays an important role in the regulation of ooctye maturation [33–35]. In this study, the enrichment of cation transmembrane transport-related GO categories confirmed that a different regulation of ion-transmembrane transport exists in yak ovaries compared with cattle ovaries, but its exact function requires further study.

**Table 2:** Top 10 enrichment GO categories in the molecular functions of DGEs.

Gene Ontology term	Cluster frequency	Genome frequency	Corrected P-value
calcium ion binding	67 out of 944 genes^a^, 7.1%	634 out of 15811 genes^b^, 4.0%	0.00232
metal ion transmembrane transporter activity	46 out of 944 genes, 4.9%	379 out of 15811 genes, 2.4%	0.00237
cation channel activity	36 out of 944 genes, 3.8%	278 out of 15811 genes, 1.8%	0.00647
transmembrane transporter activity	94 out of 944 genes, 10.0%	1024 out of 15811 genes, 6.5%	0.01108
glycosaminoglycan binding	26 out of 944 genes, 2.8%	176 out of 15811 genes, 1.1%	0.01124
monovalent inorganic cation transmembrane transporter activity	42 out of 944 genes, 4.4%	355 out of 15811 genes, 2.2%	0.01168
cation transmembrane transporter activity	61 out of 944 genes, 6.5%	592 out of 15811 genes, 3.7%	0.01386
gated channel activity	37 out of 944 genes, 3.9%	301 out of 15811 genes, 1.9%	0.01587
inorganic cation transmembrane transporter activity	53 out of 944 genes, 5.6%	498 out of 15811 genes, 3.1%	0.02024
carbohydrate derivative binding	26 out of 944 genes, 2.8%	187 out of 15811 genes, 1.2%	0.03270

a. The number of differentially expressed genes with biological processes with GO annotation.

b. The number of genes in the entire yak genome with biological processes with GO annotation.

In the cellular component category, 427 GO categories were enriched, the top 10 of which are shown in Table 3. Among the top ten enriched GO categories, all categories belong to the extracellular or plasma membrane parts. The result is consistent with those in the biological processes and molecular function categories. For example, the adhesion-related categories, hormone-related categories, and cation transmembrane transport-related categories occurred in the extracellular or the plasma membrane parts. These results further confirm the accuracy of the enrichment.

**Table 3:** Top 10 enrichment GO categories in the cellular components of DGEs.

Gene Ontology term	Cluster frequency	Genome frequency	Corrected P-value
extracellular region	209 out of 1054 genes^a^, 19.8%	2000 out of 17161 genes^b^, 11.7%	7.08e-13
extracellular region part	126 out of 1054 genes, 12.0%	1096 out of 17161 genes, 6.4%	1.32e-09
plasma membrane part	182 out of 1054 genes, 17.3%	1923 out of 17161 genes, 11.2%	3.74e-07
extracellular space	95 out of 1054 genes, 9.0%	856 out of 17161 genes, 5.0%	4.77e-06
cell periphery	332 out of 1054 genes, 31.5%	4213 out of 17161 genes, 24.5%	3.42e-05
intrinsic to plasma membrane	116 out of 1054 genes, 11.0%	1202 out of 17161 genes, 7.0%	0.00024
plasma membrane	318 out of 1054 genes, 30.2%	4109 out of 17161 genes, 23.9%	0.00045
cell surface	59 out of 1054 genes, 5.6%	506 out of 17161 genes, 2.9%	0.00065
integral to plasma membrane	111 out of 1054 genes, 10.5%	1164 out of 17161 genes, 6.8%	0.00078
extracellular matrix	50 out of 1054 genes, 4.7%	427 out of 17161 genes, 2.5%	0.00364

a. The number of differentially expressed genes with biological processes with GO annotation.

b. The number of genes in the entire yak genome with biological processes with GO annotation.

**Fig 1:**
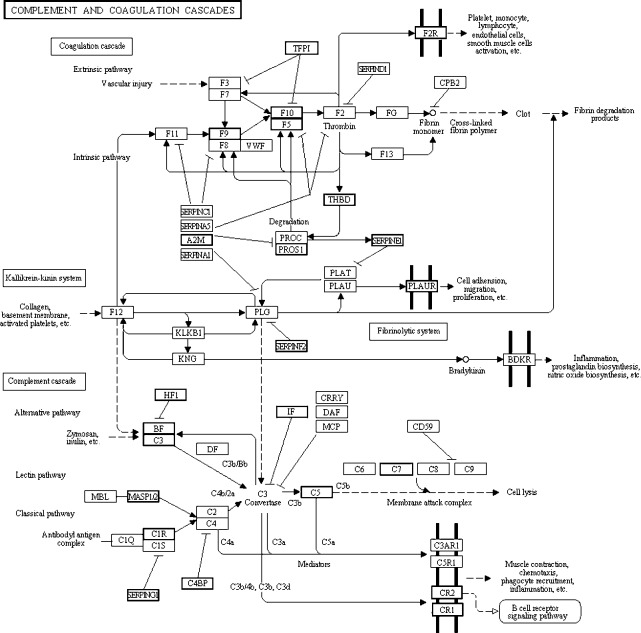
Differentially expressed genes in the complement and coagulation cascades pathway. Bold boxes indicate the genes that were differentially expressed. The upregulated genes including: THBD, A2M, PROS1, SERPNE1, PLAUR, SERPNF2, IF, BF, C3, C5, C7, MASP1/2, C1R, C4BP, SERPNG1, CR2, CR1; The downregulated genes including:F2R, TFP1, F9, F10; The genes family members has both upregulated and downregulated including: F5, HF1.

KEGG enrichment analysis showed that 243 pathways were enriched in DEGs, and the top 10 of which are shown in Table 4. Among the pathways, the “complement and coagulation cascades” pathway was the most enriched. The complement is a set of heat-labile globulin found in tissue fluids with enzyme activity, which plays an important role in innate and adaptive immunity [36]. The complement triggers a molecular effect through three kinds of cascade pathways, namely, classical, lectin, and bypass, which activate a series of cascade responses. The activated complement can regulate phagocytes and immune cells and enhance inflammatory response; it can also kill certain bacteria or cells directly by dissolving cytoderm [37]. Blood coagulation is a process by which fibrinogen in plasma is transformed into insoluble fibrin by extrinsic and intrinsic coagulation cascade pathways [38]. The “complement and coagulation cascades” pathway is a type of endogenous metabolic cascade. In addition to the main recognized physiological function, this pathway is also closely associated to many other physiological and pathological regulation processes. For example, recent studies have shown that the complement system plays an important role in many biological processes, including reproduction, development, stem cell differentiation, and tissue regeneration [39, 40]. The Bf and DAF factors in the complement system play an important role in follicular development and maturation of ovules and gametes [41]. As shown in Fig 1, the expression levels of key genes that activate complement in three kinds of cascade ways were all increased. This result suggests that the downstream physiological function including cell lysis, muscle contraction, chemotaxis, phagocyte recruitment, and inflammation could be strengthened. In the coagulation cascade pathway, we found that the genes in the extrinsic pathway that activate coagulation were only slightly expressed. This suggests that the main physiological functions including vascular injury, fibrin degradation products, and nitric oxide biosynthesis could be reduced. However, the genes in the intrinsic pathway were highly expressed, suggesting that the related downstream physiological functions, including inflammation and cell adhesion, could be strengthened. The strengthening of the “cell adhesion” progress is consistent with the enrichment results in the biological processes of the GO category, confirming that this progress is an important difference between the physical activities of yak and cattle ovaries. This result indicates that the “complement and coagulation cascades” pathway is important in the physiological activity of yak ovary, where it can regulate immunity, inflammation, reproduction, and development. In addition, we speculate that this pathway may be related to the special environment that yaks live in. To resist ovarian damage and dysfunction caused by harsh plateau conditions, the yak may have formed stronger molecular defense mechanisms than cattle.

**Table 4:** Top 10 enrichment KEGG pathways in the DGEs.

	Pathway	DEGs with pathway annotation (1157) ^a^	All genes with pathway annotation (18965)^b^	P-value
1	Complement and coagulation cascades	31 (2.68%)	206 (1.09%)	2.910213e-06
2	Amoebiasis	83 (7.17%)	938 (4.95%)	0.0003921369
3	Arrhythmogenic right ventricular cardiomyopathy (ARVC)	20 (1.73%)	145 (0.76%)	0.0005192677
4	ECM–receptor interaction	66 (5.7%)	720 (3.8%)	0.0006085478
5	Glycerolipid metabolism	14(1.21%)	89(0.47%)	0.0009503658
6	Metabolism of xenobiotics by cytochrome P450	15 (1.3%)	117 (0.62%)	0.005011024
7	Phagosome	34 (2.94%)	366 (1.93%)	0.009756936
8	Protein digestion and absorption	60 (5.19%)	729 (3.84%)	0.01112557
9	Drug metabolism—cytochrome P450	12 (1.04%)	94 (0.5%)	0.01174199
10	Circadian rhythm—mammal	6 (0.52%)	36 (0.19%)	0.02055234

a. The number of differentially expressed genes with pathway annotation.

b. The number of genes in the entire yak genome with pathway annotation.

Among the top 10 pathways, “metabolism of xenobiotics by cytochrome P450” and “drug metabolism-cytochrome P450” were also enriched. Cytochrome P450 is a type of heme-thiolate protein super family that mainly includes CYP1, CYP2, and CYP3 subfamilies. It is named based on its combination with CO and a maximum absorption peak in the 450-nm wavelength. Cytochrome P450 is an important metabolic enzyme system that can catalyze a variety of oxidation reactions in endogenous and exogenous compounds in the body, change the active or toxic compounds in chemicals, and alter drug efficacy. Cytochrome P450 is involved in the biotransformation of exogenous substances, associated with the metabolism of endogenous substances, and is an important compound in the metabolic regulation of the body [42]. Moreover, recent studies have shown that cytochrome P450 takes part in the biosynthesis of the sterol and steroid hormone, especially the sex hormone, and plays an important role in the regulation of reproductive hormones [43, 44]. This result is consistent with the enrichment of hormone-related GO categories in the biological processes, confirming that different hormone regulation systems exist between yak and cattle ovaries. In addition, part of the study demonstrated that hypoxia, including high-altitude hypoxia, can significantly change the gene expression levels in cytochrome P450 subfamilies. Hypoxia-inducible factor 1 (HIF-1), an important factor that regulates hypoxia-resistance genes in response to low oxygen, is more concerned with high-altitude hypoxia regulation. The study found that CYP3A6 promoter, a cytochrome P450 member, can combine with HIF-1 gene specificity and transactivation to induce low-oxygen resistance gene expression [45]. Moreover, cytochrome P450 members CYP17A1 and CYP2E1 genes are closely associated with the Tibetan people, helping them adapt to the plateau’s low oxygen environment [46]. Yaks have been living in the Qinghai–Tibet Plateau for a long time. Because two cytochrome P450 pathways were significantly enriched in this study, we speculate that this may also be associated with the yak’s adaptability to the plateau’s hypoxic environment.

The “ECM–receptor interaction” pathway was also enriched. The ECM is a complex matrix of biological macromolecules, including glycoproteins, protein polysaccharides, and amino sugars. The ECM has an important function in various aspects of cell physiological activities, such as cell adhesion, movement, proliferation, and differentiation, through interactions with its surface ECM receptor. For example, the ECM can transfer signals to cells via surface receptors during cell adhesion; moreover, through various signaling transduction pathways, the ECM can send signals to the cytoplasm and nucleus to influence gene expression or cellular activities [47]. In the present study, transcripts associated with the “ECM–receptor interaction” pathway were differentially expressed. This result suggests that these transcripts may be complementary to the enrichment of cell adhesion-related GO categories in biological processes and may have important functions in promoting cell adhesion and connection. The results from the present study confirmed that adhesion and connection mechanisms are important in the physiological activity of ovaries at the molecular level. Moreover, the cell connection and adhesion mechanism may be different between yak and cattle ovary.

**Fig 2:**
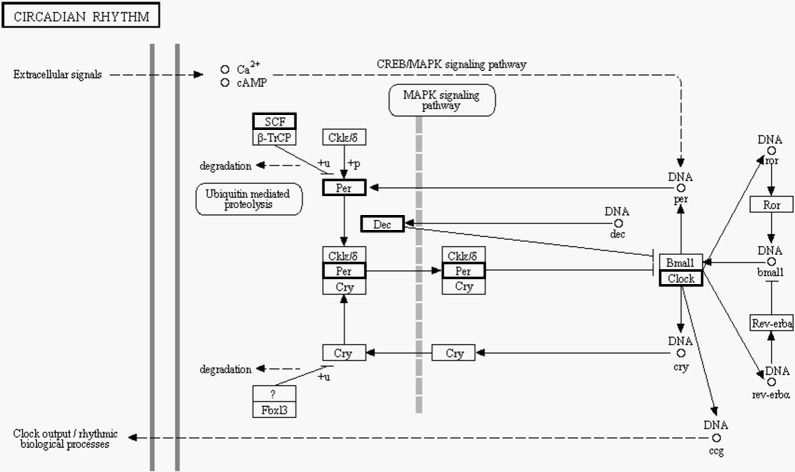
Differentially expressed genes in the circadian rhythm pathway. Bold boxes indicate the genes that were differentially expressed. The upregulated genes including: DEC; The downregulated genes including: CLOCK; The genes family members has both upregulated and downregulated including: SCF, PER.

Interestingly, several novel pathways that have no significant associations with the reproductive function of the ovary were also enriched in the top 10, such as the “circadian rhythm” pathway. The physiological and metabolic activities and behavior of different organisms follow a circadian rhythm, which is a special mechanism referred to as “biological clock.” The molecular basis of this mechanism depends on the coordinate expression of related biological clock genes; the produced clock proteins form a negative transcription–translation feedback loop, which maintains the circadian rhythm and helps mammals adapt to environmental changes [48]. The main identified clock proteins in mammals include CLOCK (circadian locomotor output cycle kaput) protein, BMALL (brain and muscle ARNT-like protein 1) protein, CRY (cryptochrome) family, and PER (period) family, wherein the CLOCK and BMALL proteins are at the core of circadian rhythm regulation [49]. In the present study, the “circadian rhythm” pathway was enriched and the expression of the CLOCK gene was downregulated in yak compared with cattle data (Fig 2). We speculate that this may be associated with the special environment that yaks live in. Because of the high altitude, hypoxic environment, and strong ultraviolet rays at the Qinghai–Tibet Plateau, the yak is more susceptible to the weak oscillation of environmental conditions, such as temperature and light. Their reproduction, which shows in the typical seasonal breeding, is also more susceptible to the limitations of seasonal change. However, further studies are needed to determine the exact functions of the pathways in the yak ovary.

## Conclusions

In conclusion, our study aimed to understand the molecular mechanism in yak ovary and the specificity of yak reproduction. After deep sequencing, a yak ovary library with 3,653,032 clean reads with a total of 4,828,772,880 bp were obtained. Alignment analysis showed that 16992 yak genes mapped to the yak genome. Of these, 12,731 and 14,631 genes were assigned to GO categories and KEGG pathways. Further by comparing the estrus ovary transcriptomes of yak and cattle, we identified 1307 genes that were differentially expressed significantly between the two libraries. Further functional analysis showed that these differences were involved in many biological events. GO categorical analysis showed that the “cell adhesion” and “hormone”-related categories were the most enriched biological processes, whereas the “calcium ion binding” and “cation transmembrane transport”-related categories were the most enriched molecular events. From the KEGG pathway analysis, the “complement and coagulation cascades” pathway was the most enriched, followed by two cytochrome P450 pathways and the “ECM–receptor interaction” pathway, which is consistent with the related GO category enrichment results. Interestingly, the “circadian rhythm” pathway was also enriched, despite having no evident associations with the reproductive function of the ovary. Our results serve as a basis to explore the molecular mechanisms of yak ovary further and may provide new insights to understand the specificity of yak reproduction.

## Supporting Information

S1 Fig GO function classification of the yak estrus ovary transcriptome. (TIF)

S1 Table Comparison of expression patterns between RNA-seq expression and qPCR. (XLSX)

S2 Table Variation detection between yak and cattle group members. (DOCX)

S3 Table Top 10 KEGG enrichment pathways in the yak estrus ovary transcriptome. (DOCX)
