# Supplementary material for: Toward Understanding the Genetic Basis of Yak Ovary Reproduction: A Characterization and Comparative Analyses of Estrus Ovary Transcriptiome in Yak and Cattle
Source: PLoS One. 2016 Apr 4;11(4):e0152675. doi: 10.1371/journal.pone.0152675 (PMC4820115; doi:10.1371/journal.pone.0152675)
Supplement: S2 Table — (DOCX) [file pone.0152675.s003.docx]

**Table S2.** Variation detection between yak and cattle group members.

|  | Variation in the yak group(n=3) | | | Variation in the cattle group（n=3） | | |
| --- | --- | --- | --- | --- | --- | --- |
| Gene name | Average CT value | Standard deviation(S.D) | Coefficient of variation (CVs)(%) | Average CT value | Standard deviation(S.D) | Coefficient of variation (CVs)(%) |
| Estrogen receptor beta(ERβ) | 20.16 | 0.31 | 1.52 | 18.13 | 0.37 | 2.05 |
| Complement Factor B(BF) | 17.55 | 0.33 | 1.88 | 19.18 | 0.25 | 1.28 |
| Homeobox protein Hox-B4(HOXB4) | 16.13 | 0.24 | 1.51 | 21.57 | 0.36 | 1.68 |
| clock circadian regulator (CLOCK) | 22.31 | 0.36 | 1.62 | 19.46 | 0.23 | 1.19 |
| Cytochrome P450 3A24 (CYP3A24) | 16.53 | 0.22 | 1.33 | 20.23 | 0.31 | 1.55 |
