# Supplementary material for: Toward Understanding the Genetic Basis of Yak Ovary Reproduction: A Characterization and Comparative Analyses of Estrus Ovary Transcriptiome in Yak and Cattle
Source: PLoS One. 2016 Apr 4;11(4):e0152675. doi: 10.1371/journal.pone.0152675 (PMC4820115; doi:10.1371/journal.pone.0152675)
Supplement: S3 Table — (DOCX) [file pone.0152675.s004.docx]

**Table S3.** Top 10 KEGG enrichment pathways in the yak estrus ovary transcriptome.

|  | Pathway | Expressed genes with pathway annotation (14,631) ^a)^ | All genes with pathway annotation (18,965) ^b)^ | *P* value |
| --- | --- | --- | --- | --- |
| 1 | [Focal adhesion](file:///E:\%E9%AB%98%E5%8E%9F%E5%9B%BE%E7%89%87\%E5%8D%B5%E5%B7%A2%E6%9C%80%E6%96%B0%E5%88%86%E6%9E%90\Kegg_All\MaoNiuLuanChao.html#gene1) | 893 (6.1%) | 985 (5.19%) | 5.924522e-33 |
| 2 | [Pathways in cancer](file:///E:\%E9%AB%98%E5%8E%9F%E5%9B%BE%E7%89%87\%E5%8D%B5%E5%B7%A2%E6%9C%80%E6%96%B0%E5%88%86%E6%9E%90\Kegg_All\MaoNiuLuanChao.html#gene2) | 559 (3.82%) | 608 (3.21%) | 9.409632e-23 |
| 3 | [ECM-receptor interaction](file:///E:\%E9%AB%98%E5%8E%9F%E5%9B%BE%E7%89%87\%E5%8D%B5%E5%B7%A2%E6%9C%80%E6%96%B0%E5%88%86%E6%9E%90\Kegg_All\MaoNiuLuanChao.html#gene3) | 652 (4.46%) | 720 (3.8%) | 9.650817e-22 |
| 4 | [Amoebiasis](file:///E:\%E9%AB%98%E5%8E%9F%E5%9B%BE%E7%89%87\%E5%8D%B5%E5%B7%A2%E6%9C%80%E6%96%B0%E5%88%86%E6%9E%90\Kegg_All\MaoNiuLuanChao.html#gene4) | 828 (5.66%) | 938 (4.95%) | 3.527666e-19 |
| 5 | [Regulation of actin cytoskeleton](file:///E:\%E9%AB%98%E5%8E%9F%E5%9B%BE%E7%89%87\%E5%8D%B5%E5%B7%A2%E6%9C%80%E6%96%B0%E5%88%86%E6%9E%90\Kegg_All\MaoNiuLuanChao.html#gene5) | 576 (3.94%) | 655 (3.45%) | 6.055588e-13 |
| 6 | [Axon guidance](file:///E:\%E9%AB%98%E5%8E%9F%E5%9B%BE%E7%89%87\%E5%8D%B5%E5%B7%A2%E6%9C%80%E6%96%B0%E5%88%86%E6%9E%90\Kegg_All\MaoNiuLuanChao.html#gene6) | 282 (1.93%) | 306 (1.61%) | 2.021385e-12 |
| 7 | [MAPK signaling pathway](file:///E:\%E9%AB%98%E5%8E%9F%E5%9B%BE%E7%89%87\%E5%8D%B5%E5%B7%A2%E6%9C%80%E6%96%B0%E5%88%86%E6%9E%90\Kegg_All\MaoNiuLuanChao.html#gene12) | 303 (2.07%) | 332 (1.75%) | 8.067557e-12 |
| 8 | [Endocytosis](file:///E:\%E9%AB%98%E5%8E%9F%E5%9B%BE%E7%89%87\%E5%8D%B5%E5%B7%A2%E6%9C%80%E6%96%B0%E5%88%86%E6%9E%90\Kegg_All\MaoNiuLuanChao.html#gene8) | 406 (2.77%) | 455 (2.4%) | 1.426242e-1 |
| 9 | [Insulin signaling pathway](file:///E:\%E9%AB%98%E5%8E%9F%E5%9B%BE%E7%89%87\%E5%8D%B5%E5%B7%A2%E6%9C%80%E6%96%B0%E5%88%86%E6%9E%90\Kegg_All\MaoNiuLuanChao.html#gene9) | 228 (1.56%) | 245 (1.29%) | 1.784865e-11 |
| 10 | [Wnt signaling pathway](file:///E:\%E9%AB%98%E5%8E%9F%E5%9B%BE%E7%89%87\%E5%8D%B5%E5%B7%A2%E6%9C%80%E6%96%B0%E5%88%86%E6%9E%90\Kegg_All\MaoNiuLuanChao.html#gene10) | 264 (1.8%) | 288 (1.52%) | 5.144035e-1 |

a) The number of expressed genes in yak ovary with pathway annotation.

b) The number of genes in the whole yak genome with pathway annotation.
